# Path analysis of the association between self-compassion and depressive symptoms among nursing and medical students: a cross-sectional survey

**DOI:** 10.1186/s12912-022-00835-z

**Published:** 2022-03-24

**Authors:** Fang-Fang Zhao, Li Yang, Jiang-Ping Ma, Zheng-Ji Qin

**Affiliations:** 1 Department of Nursing Science, School of Medicine, Nantong University, NantongJiangsu Province, 0086-226001 China; 2 School of Nursing Science, Faculty of Medicine, Qingdao University, Qingdao CityShandong Province, 0086-266021 China; 3 Department of Nursing Science, ChangZhi Medical College, Changzhi CityShanxi Province, 0086-046000 China; 4 Department of Epidemiology and Statistics, School of Public Health, Nantong University, NantongJiangsu Province, 0086-226001 China

**Keywords:** Depressive symptoms, Self-compassion, Resilience, Optimism, Perceived stress, College students

## Abstract

**Background:**

Nursing and medical students are suffering from high rates of depressive symptoms. Mental health benefits students’ learning, growth and professional development. Exploring psychological resources to prevent depression is emphasized recently, and self-compassion is shown to be inversely associated with depressive symptoms. However, the mechanism through which self-compassion contributes to decreased depressive symptoms is limited. Therefore, this study aimed to explore and examine a model detailing the potential paths between self-compassion and depressive symptoms.

**Methods:**

A cross-sectional study was conducted and convenient sampling was used. Among the 1800 nursing and medical students targeted from two universities in East and North China, 1341 completed the questionnaires, and 1127 valid questionnaires were analyzed comprising 566 and 561 from medical and nursing students, respectively. Data in May 2020 and July 2020 were collected through Patient Health Questionnaire, self-compassion scale, resilience scale, Life Orientation Test and Perceived Stress Scale. Then, path model analysis was conducted to analyze the data.

**Results:**

Finally, this study included 1125 valid questionnaires after excluding two extremes of study variables. Participants consisted of 50.2% medical students and 49.8% nursing students. The model showed an acceptable fit to the data. After controlling for the demographics, self-compassion was directly and indirectly associated with decreased depressive symptoms by increasing resilience and optimism and reducing perceived stress among nursing and medical students. Resilience and optimism were directly and indirectly associated with decreased depressive symptoms by reducing perceived stress among nursing students and indirectly associated with decreased depressive symptoms among medical students.

**Conclusions:**

The study provides evidence that self-compassion significantly influences the decrease in depressive symptoms by increasing resilience and optimism and reducing perceived stress. These findings suggested that programs enhancing students’ self-compassion, resilience, and optimism simultaneously can help decrease depressive symptoms and improve mental health in education and healthcare institutes. These findings may facilitate the designing of educational programs for preventing depressive symptoms and promoting mental health among nursing and medical students.

## Background

Nursing and medical students experience high rates of depressive symptoms [1, 2]. The overall pooled crude prevalence of depression or depressive symptoms among medical and nursing students was 27.2% [3] and was 34.0% [4], respectively. However, approximately half of the studies that used popularly digital mental health interventions in dealing with depression did not show the expected outcomes [5]. Moreover, when students had mental distress, many of them are likely to solve the mental health problem without treatment [6]. Combined with the lack of mental health professionals, the treatment rate is low, for example, in many countries, less than 10% of people of all ages received treatment [7].

Whereas mental health is important for students to contribute fully to their learning and growth [8]. Mentally health nursing and medical students tend to be productive and successful in their academic performance and clinical studies and provide quality patient care [1, 9, 10]. Identifying the factors that can prevent and reduce depressive symptoms can provide information for designing effective training programs that help nursing and medical students to improve their mental health.

## Self-compassion

Negative life events increased the risk of depression [11]. Nevertheless, certain individuals perceive less stress from negative life events and can positively adapt to adversities more successfully compared with those who suffered mental disorders [12]. Such findings also promote researchers to explore the psychological resources that influence the processes of responding to stressors. Self-compassion has been recently shown inverse relationship with depressive symptoms [13]. From the Buddhist philosophy, self-compassion including three key components referring to self-kindness, common humanity, and mindfulness, is characterized by the gentleness with oneself when facing hardships [14]. Self-compassion helps people treat negative thoughts and emotions as a common humanity and in nonjudgmental awareness, not ruminate on them [15]. Thus, self-compassionate people are likely to reduce distress [16].

Certain studies have examined the possible mediators between self-compassion and depressive symptoms. For example, the self-compassion–depressive symptoms relationship was mediated by emotion regulation strategies, including rumination, avoidance, and acceptance among adults with recurrent depression (aged 21 – 66 years) [17], the ability to tolerate negative emotions among clinically depressed individuals [18], rumination among first-year psychology undergraduates [19] and breast cancer survivors [20] and perceived stress among medical workers [21].

These previous studies explored the mediators from emotion regulation in different groups and also mentioned perceived stress among medical workers. These findings laid the foundation that self-compassion is not related to depressive symptoms alone and only directly. In addition to emotion regulation skills, other variables, such as positive coping skills, and attitudes, such as resilience and optimism, should be considered.

## Resilience, optimism and perceived stress as potential mediators

Resilience refers to the ability of individuals to recover from a negative experience and difficult situation. Resilience showed an inverse association with depression among middle and high schools adolescents [11] and older adults [22]. Extrapolating these findings to nursing and medical schools may not be the same as the college period involves different psychological changes, especially amid the COVID-19 pandemic when social expectations have changed healthcare services. Researchers have focused on the association between resilience and depressive symptoms. Thus, neglecting resilience may decrease depressive symptoms by the mediation of perceived stress. Moreover, self-compassion is related to resilience for adults with epilepsy [23]. Thus, resilience may mediate Self-compassion–depression relationship and may be linked to depressive symptoms by reducing perceived stress.

Optimism involves future positive expectations, a positive attitude that outcomes will be in the right direction and will be desirable [24]. Self-compassionate people who often accept negative emotions non-judgmentally [15] may foster an ability to adopt a new perspective of and attitude toward negative and positive situations. Previous studies showed that self-compassion is associated with optimism among adults [25], undergraduate students [26], and elderly people [27]. Optimism has been linked to coping skills, which enable individuals to build personal resources to help reduce the negative impact of stressors [28]. Although optimism is associated with particular facets of well-being, the pattern of the association is complex [29]. Thus, optimism may also potentially mediate the association between self-compassion and depressive symptoms and may be linked to low depression by reducing perceived stress.

Self-compassionate people view negative emotions and experiences as part of being human [17] and think in a way that can reduce the negative impacts of adverse events [30]. Moreover, self–compassionate adolescents are also likely to reduce perceived stress [31]. Thus, self-compassion is also potentially associated with low depressive symptoms by reducing perceived stress among nursing and medical students.

In summary, the review of the literature revealed certain important gaps. Although self-compassion is gaining research attention, research about the variables that may explain (i.e. mediate) the relationships between self-compassion and depression is comparatively limited. Other variables may account for the benefits of self-compassion on decreased depression. Studies have found links to support the possible connections between self-compassion, resilience, optimism, perceived stress and depressive symptoms. However, these mediation pathways have not been explicitly tested. Moreover, how these variables function simultaneously is unknown. Hence, identifying these mediation paths between self-compassion and depressive symptoms will address the literature gaps and potentially expand the understanding of how self-compassion may translate into decreased depressive symptoms.

Both nursing and medical students experience high rates of depressive symptoms [1, 2]. Their emotional state and associated factors after the COVID-19 pandemic must be examined. Thus, both the two groups from medical schools are selected as the target sample in the study.

## Methods

### Aims

The study aimed to test the following hypotheses: H (Hypothesis).

**Fig. 1:**
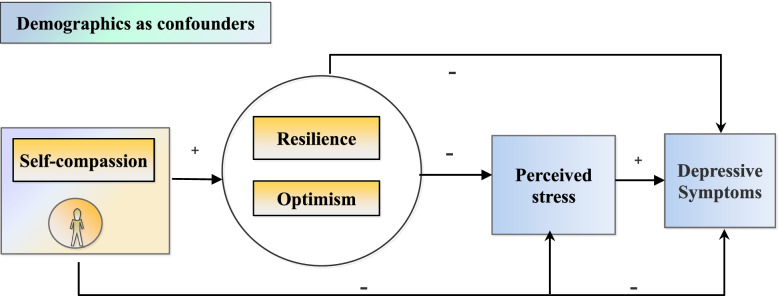
Hypothesized path model of study variables drawn in the present study Note: + and – denote positive and negative relationships, respectively

- H1: Resilience, optimism and perceived stress mediate the association between self-compassion and depressive symptoms among nursing and medical students.
- H2: Resilience and optimism are directly and indirectly associated with depressive symptoms though decreased perceived stress among medical and nursing students. Figure 1 below presents the conceptual framework. Demographics were controlled for potential confounders.

### Design

The study used a web-based survey with a cross-sectional design, which was the most appropriate approach to collect data from populations in different locations.

### Participants

Convenience sampling was conducted on data from May 2020 to July 2020 to recruit medical and nursing students from two universities in Jiangsu Province and Shanxi Province in East and North China, respectively. The nursing students were in a four-year undergraduate program, whereas the medical students were in a five-year undergraduate program. According to the *N:q* rule [32], the recommended ratio of sample size (*N*) to parameters that require statistical estimates (*q*) was 20:1. In this study, the number of potential path parameters was 24, which required a minimum sample size of 480 for each group. Considering the response rate, the target sample size totaled 1800 nursing and medical students. Thus, questionnaires were distributed to approximately 1800 nursing and medical students in two groups.

### Ethical considerations

The study was approved by the ethics review committee of Nantong university in East China (2019–17). The student participates were informed about the study, and the participation was voluntary. Consent was given verbally and included in the questionnaires submitted at their free time. Confidentiality, anonymity, and freedom to withdraw were ensured. Approval of the original authors of the instruments used in the study was obtained by e-mail when necessary. The participants were coded to avoid identifiable information before the data analysis.

## Measures

The measures included age, gender, grade and with gender  and grade as confounders, and instruments listed as follows.

### Patient Health Questionnaire depression scale

Depressive symptoms were measured by the Patient Health Questionnaire (PHQ-9), which has nine items. The scale was measured over the last 2 weeks how often individuals were bothered by symptoms with responses scored from 0 (not at all) to 3 (nearly every day). The higher the score, the more severe are the depression symptoms. Minimal, mild, moderate, moderately severe, and severe depression 1–4, 5–9, 10–14, 15–19, and 20–27, respectively [33]. The PHQ-9 has been validated in different groups and demonstrated high internal reliability with a Cronbach’s α > 0.8 [33, 34] and was 0.95 in this study.

### 10-item Connor-Davidson Resilience Scale

Resilience was measured by 10-item Connor-Davidson Resilience Scale adapted [35] from the 25-item version [36]. The 10-item resilience scale is scored on a 5-point Likert scale from 0 (never) to 4 (almost always). The Cronbach’s α was 0.85 [35] and 0.95 in this study. The higher the score, the higher is the resilience level.

### The Life Orientation Test

The Life Orientation Test (LOT) measures the individuals’ optimism levels. The LOT-R is a short version of the LOT [37] modified from the original one [24]. It is a 5-point Likert scale with options ranging from 0 (I disagree a lot) to 4 (I agree a lot). The LOT-R has 10 items with items 2, 5, 6, and 8 are filters, and the other six items are evaluated in the test. Internal consistency was acceptable with a Cronbach’s α 0.69 at baseline and 0.72 at follow up [38] and 0.67 in Chinese students [10]. A high score indicates a high optimism level.

### Self-Compassion Scale

The self-compassion scale [14] measures how individuals treated themselves when in misery. The short version of the 12-item self-compassion scale is a 5-point Likert scale with responses from 1 (almost never) to 5 (almost always) [39]. It has six dimensions, including self-kindness, common humanity, mindfulness, self-judgment, isolation, and over-identified. Self-judgment, isolation, and over-identified were reverse-scored. The scale has a Cronbach's α ≥ 0.82 [39, 40] and negatively-worded and positively worded items has a Cronbach’ α of 0.71–0.91 in this study. A higher score indicates a high self-compassion level.

### Perceived Stress Scale

The perceived Stress Scale consists of 10 items for measuring the degree of stressful situations perceived during the past month [41]. The response options of the scale range from 0 (never) to 4 (very often). Total scores are computed by summing reversing responses to the four positively stated items and other items. The Cronbach’s α of the Chinese version was 0.86 [42]. A high score indicates severer perceived stress.

### Data collection

University counselors and the researchers distributed the online self-reported questionnaire to the students after a short instruction via a survey link using the online survey tool Wenjuan Xing (Changsha Ranxing Information Technology Co., Ltd., China). Students read the study aims and instructions before deciding to answer the questionnaires. If they agreed to participate, they clicked to complete the questionnaires and submitted in their free time, clicked quitting otherwise. Each IP address was set for submitting once to avoid repeated submissions. A total of 1341 students from a university in Jiangsu Province and a college in Shanxi Province participated and filled out the questionnaires. A total of 566 questionnaires were filled out by medical students and 561 questionnaires filled out by nursing students, after excluding the quick clicks according to the completion time.

### Data analysis

The data analysis was conducted using IBM SPSS 25.0 and IBM SPSS Modeler 18.0. Data should be cleaned before analysis [43]. In IBM SPSS Modeler 18.0, outliers were adjusted to their closest normal value and extremes were discarded. Outliers referred to outside the fence of Q1 – (1.5 × Interquartile range [IQR]) and Q3 + (1.5 × IQR) and extremes outside the fence of Q1 – 3 × IQR and Q3 + 3 × IQR. Eleven students filled period in the age variable and the missing values were imputed by the mode of the ages in the same college year. The school systems among nursing (four-year system) and medical students (five-year system) have certain differences. Thus, the data were separated for analysis. The differences in characteristics of depressive symptoms were compared using the independent t-test and one-way ANOVA test. Then, the Pearson correlation analysis was conducted. The significant demographics were controlled for confounders.

Furthermore, path analysis was conducted in AMOS23.0 to test the mediating effects of optimism, resilience, and perceived stress on the relationship between self-compassion and depressive symptoms. Model fit indices Bentler’s comparative fit index (CFI), Bentler–Bonett normed fit index (NFI), Tucker–Lewis index (TLI) with indices ≥ 0.95 indicate good fitting models, whereas indices ≥ 0.90 suggest acceptable fit. Standardized root mean square residual (SRMR) and root mean square error of approximation (RMSEA) indices ≤ 0.08 indicate acceptable model fit, whereas values ≤ 0.05 indicate good model fit [44]. The 95% CI did not include 0, indicating a significant difference.

Sensitivity analyses were conducted by not dealing with and dealing with these outliers, extremes and missing values separately. Although a slight difference exists, the mediating effects of optimism, resilience, and perceived stress on the relationship between self-compassion and depressive symptoms are significant and not sensitive to the outliers and extremes.

## Results

### Participant characteristics

Among the 1127 questionnaires, 1125 were considered valid and finally included in this study after excluding two extremes of variables (one extreme in self-compassion and one in optimism). The data set for analysis comprised 565 medical students and 560 nursing students, of which 30.4% and 9.8% were males respectively. The average age in the nursing students was 20.75 (SD:1.37; range:17–24 years) and was 20.83 (SD:1.80; range:17–29 years) in medical students. The mean score of depressive symptoms among the nursing students was 6.93 (SD: 5.46) and was 6.31 (SD: 5.58) among medical students. Among the total students, 18.8% had scored PHQ ≥ 10, with 19.6% among nursing students and 18.1% among medical students. Table 1 below presents the means and SD scores of the study variables.

**Table 1:** Participants' demographic information and comparisons of study variables of nursing and medical students

	**N (%)**	**Depressive symptoms**	***p***	**Self-compassion**	***p***	**Resilience**	***p***	**Optimism**	***p***	**Perceived stress**	***p***
**Nursing students**											
**Gender**			0.265		0.694		0.104		0.080		0.219
Male	55 (9.8)	7.71 ± 5.99		37.41 ± 4.35		27.38 ± 6.62		12.43 ± 2.02		18.27 ± 3.55	
Female	505 (90.2)	6.84 ± 5.40		37.61 ± 3.44		26.00 ± 5.89		12.92 ± 1.96		17.62 ± 3.76	
**College year(Grade)**			**0.048**		0.862		0.505		0.347		0.142
1^st^ year	140 (25.0)	6.26 ± 5.15		37.64 ± 3.49		26.46 ± 5.73		12.96 ± 2.06		17.37 ± 3.90	
2^nd^ year	193 (34.5)	7.80 ± 5.67		37.44 ± 3.41		25.78 ± 6.24		12.71 ± 1.71		18.16 ± 3.37	
3^rd^ year	91(16.3)	6.49 ± 5.37		37.56 ± 3.83		25.73 ± 5.63		12.80 ± 2.08		17.24 ± 3.87	
4^th^ year	136 (24.3)	6.66 ± 5.45		37.77 ± .3.59		26.60 ± 6.06		13.08 ± 2.14		17.63 ± 3.97	
Total	560	6.93 ± 5.46		37.59 ± 3.54		26.14 ± 5.97		12.88 ± 1.97		17.68 ± 3.74	
**Medical students**											
**Gender**			0.592		0.237		** < 0.001**		0.213		0.137
Male	172 (30.4)	6.51 ± 6.12	0.605	38.02 ± 3.91		30.36 ± 7.31		13.00 ± 2.43		17.38 ± 4.48	
Female	393 (69.6)	6.22 ± 5.34		38.47 ± 4.30		27.72 ± 6.68		13.28 ± 2.47		16.75 ± 4.63	
**College year**											
1^st^ year	200 (35.4)	5.23 ± 4.92	** < 0.01**	37.99 ± 3.87	0.272	28.08 ± 6.98	0.467	13.04 ± 2.42	0.487	16.81 ± 4.45	0.795
2^nd^ year	139 (24.6)	7.54 ± 6.33		38.21 ± 4.01		28.20 ± 7.05		13.05 ± 2.42		17.34 ± 4.94	
3^rd^ year	79 (14.0)	6.88 ± 6.18		38.22 ± 4.43		29.11 ± 7.05		13.41 ± 2.45		17.02 ± 4.68	
4^th^ year	41 (7.3)	6.02 ± 4.45		38.94 ± 4.31		28.27 ± 6.74		13.44 ± 1.90		16.64 ± 4.99	
5^th^ year	106 (18.8)	6.39 ± 5.35		39.01 ± 4.72		29.44 ± 6.93		13.45 ± 2.77		16.71 ± 4.20	
Total	565	6.31 ± 5.58		38.33 ± 4.19		28.52 ± 6.98		13.20 ± 2.46		16.94 ± 4.59	

The Table 1 summarizes the demographics and their association with depressive symptoms, self-compassion, resilience, optimism, and perceived stress. Gender is associated with resilience among medical students. The one-way ANOVA test shows that college year is associated with depressive symptoms among nursing and medical students.

### Pearson’s correlation analysis

Table 2 illustrates that self-compassion among the nursing students is positively associated with resilience (*r* = 0.400, *P* < 0.01) and optimism (*r* = 0.449, *P* < 0.01) and negatively associated with perceived stress (*r* =  − 0.445, *P* < 0.01) and depressive symptoms (*r* =  − 0.365, *P* < 0.01). In contrast, self-compassion among medical students, is positively associated with resilience (*r* = 0.438, *P* < 0.01) and optimism (*r* = 0.623, *P* < 0.01) and negatively associated with perceived stress (*r* =  − 0.571, *P* < 0.01) and depressive symptoms (*r* =  − 0.369, *P* < 0.01).

**Table 2:** Correlations between self-compassion, resilience, optimism, perceived stress and depressive symptoms

Variables	Self-compassion	Resilience	Optimism	Perceived stress	Depressive symptoms
Self-compassion	1	0.438**	0.623**	− 0.571**	− 0.369**
Resilience	0.400^**^	1	0.390**	− 0.375**	− 0.289**
Optimism	0.449^**^	0.333^**^	1	− 0.524**	− 0.327**
Perceived stress	− 0.445^**^	− 0.413^**^	− 0.392^**^	1	0.474**
Depressive symptoms	− 0.365**	− 0.389**	− 0.331**	0.565**	1

Note. The nursing students are showed in the lower left diagonal matrix (*N* = 560). The medical students are showed in the upper right diagonal matrix (*N* = 565). **p < 0.01

### Path analysis

**Fig. 2:**
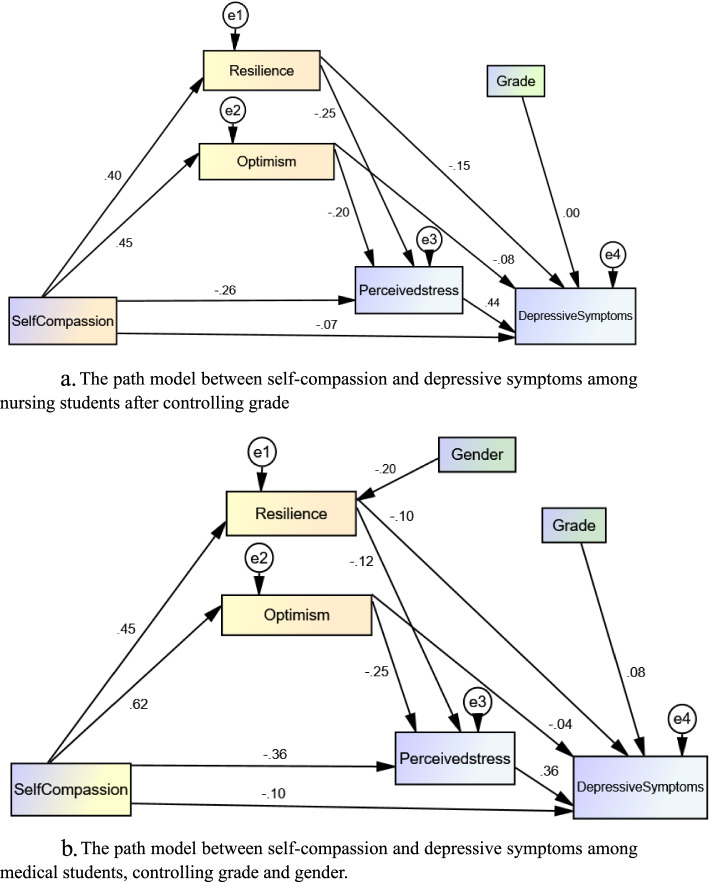
**a **The path model between self-compassion and depressive symptoms among nursing students after controlling grade. **b** The path model between self-compassion and depressive symptoms among medical students, controlling grade and gender

The path analysis was conducted to examine the mediating effects of resilience, optimism and perceived stress, controlling for grade and/or gender based on the analysis in Table 1. The model indicates acceptable fit to the data for nursing students (x^2^ = 20.495, df = 5, NFI = 0.970, TLI = 0.931, CFI = 0.977, RMSEA = 0.074, SRMR = 0.037) and medical students (x^2^ = 29.897, df = 10, NFI = 0.967, TLI = 0.952, CFI = 0.977, RMSEA = 0.059, SRMR = 0.042) (Fig. 2a, b).

Regarding nursing students, Table 3 shows that self-compassion has direct effects (β =  − 0.075; 95% CI: − 0.149, − 0.003) and indirect effects (β =  − 0.292; 95% CI: − 0.353, − 0.229) on depressive symptoms by improving resilience and optimism and decreasing perceived stress among nursing students. Self-compassion also had indirect effects on perceived stress (β =  − 0.187; 95% CI: − 0.242, − 0.134) by improving resilience and optimism. Resilience (β =  − 0.154; 95% CI: − 0.247, − 0.042) and optimism (β =  − 0.075; 95% CI: − 0.136, − 0.011) had direct effects on depressive symptoms. Resilience (β =  − 0.108; 95% CI: − 0.151, − 0.075) and optimism (β = -0.086; 95% CI: − 0.142, − 0.042) also had indirect effects on depressive symptoms through decreasing perceived stress. Table 3.

**Table 3:** Standardized total effects, direct and indirect effects of self-compassion, optimism, resilience and perceived stress

	**Total effects**	**Bias-corrected 95% CI**	**Direct** **effects**	**Bias-corrected 95% CI**	**Indirect effects**	**Bias-corrected 95% CI**
**Nursing students**						
Self-compassion→Resilience	0.400	(0.319, 0.471)	0.400	(0.319, 0.471)	NA	
Self-compassion→Optimism	0.449	(0.352,0.533)	0.449	(0.352,0.533)	NA	
Self-compassion→Perceived stress	-0.448	(-0.528, -0.354)	-0.262	(-0.360, -0.160)	-0.187	(-0.242, -0.134)
Self-compassion→Depressive Symptoms	-0.367	(-0.428, -0.291)	-0.075	(-0.149, -0.003)	-0.292	(-0.353, -0.229)
Resilience→Perceived stress	-0.246	(-0.324, -0.170)	-0.246	(-0.324, -0.170)	NA	
Resilience→Depressive Symptoms	-0.262	(-0.351, -0.132)	-0.154	(-0.247, -0.042)	-0.108	(-0.151, -0.075)
Optimism→Perceived stress	-0.196	(-0.301, -0.098)	-0.196	(-0.301, -0.098)	NA	
Optimism→Depressive Symptoms	-0.161	(-0.231, -0.098)	-0.075	(-0.136, -0.011)	-0.086	(-0.142, -0.042)
Perceived stress→Depressive Symptoms	0.439	(0.354, 0.532)	0.439	(0.354,0.532)	NA	
**Medical students**						
Self-compassion→Resilience	0.446	(0.375, 0.502)	0.446	(0.375, 0.502)	NA	
Self-compassion→Optimism	0.623	(0.547, 0.686)	0.623	(0.547,0.686)	NA	
Self-compassion→Perceived stress	-0.573	(-0.633, -0.502)	-0.362	(-0.463, -0.259)	-0.211	(-0.288, -0.139)
Self-compassion→Depressive Symptoms	-0.378	(-0.445, -0.306)	-0.103	(-0.203, -0.010)	-0.275	(-0.357, -0.189)
Resilience→Perceived stress	-0.119	(-0.197, -0.045)	-0.119	(-0.197, -0.045)	NA	
Resilience→Depressive Symptoms	-0.142	(-0.246, -0.021)	-0.100	(-0.202, 0.014)	-0.042	(-0.077, -0.017)
Optimism→Perceived stress	-0.254	(-0.347, -0.151)	-0.254	(-0.347, -0.151)	NA	
Optimism→Depressive Symptoms	-0.134	(-0.221, -0.053)	-0.043	(-0.125, 0.042)	-0.090	(-0.139, -0.052)
Perceived stress→Depressive Symptoms	0.355	(0.232, 0.463)	0.355	(0.232, 0.463)	NA	

*NA* not available

Regarding medical students, Table 3, further shows that self-compassion had direct effects (β =  − 0.103; 95% CI: − 0.203, − 0.010) and indirect effects (β =  − 0.275; 95% CI: − 0.357; − 0.189) on depressive symptoms by improving resilience and optimism and decreasing perceived stress among nursing students. Self-compassion also had indirect effects on perceived stress (β =  − 0.211; 95% CI: − 0.288, − 0.139) by improving resilience and optimism. Resilience (β =  − 0.100; 95% CI: − 0.202, 0.014) and optimism (β =  − 0.043; 95% CI: − 0.125, 0.042) had no direct effects on depressive symptoms. Resilience (β =  − 0.042; 95% CI: − 0.077, − 0.017) and optimism (β =  − 0.090; 95% CI: − 0.139, − 0.052) had indirect effects on depressive symptoms by decreasing perceived stress.

## Discussion

The present study tested a model and indicated that resilience, optimism and perceived stress mediated the association between self-compassion and depressive symptoms among nursing and medical students, thus supporting H1. Resilience and optimism were directly and indirectly associated with depressive symptoms among nursing students and indirectly associated with depressive symptoms among medical students.

Resilience mediated the relationships of self-compassion with perceived stress and with depressive symptoms among nursing and medical students in this study. This result can be explained that self-compassion contributes to environmental mastery and positive social relations with others [26]. As a result, the resilience ability to overcome the negative effects of adverse situations can be enhanced. Resilient individuals utilize personal resources, deal with the stressful encounters, and adapt to adverse situations with confidence [45]. The present study indicates that resilience is not only linked to decreased perceived stress and depressive symptoms but also has a mediating effect on the relationship of self-compassion with perceived stress and with depressive symptoms in both groups. The study highlights the importance of resilience throughout the curriculum in both groups. Individual resilience may be developed through self-compassion techniques as self-compassion and resilience are significantly associated.

Optimism mediated the relationships of self-compassion with perceived stress and with depressive symptoms among the nursing and medical students in this study. Self-compassion can foster adaptive coping strategies [26] and encourage individuals to believe positively in their ability to create an expected future. The results are consistent with research examining the positive role of self-compassion in enhancing optimism among adults [25] and undergraduate students [26]. The present study provides additional evidence that optimism enables youths to face negative life events and promotes their psychological well-being [29]. The present study also extends that self-compassion can reduce depressive symptoms by enhancing optimism among nursing and medical students.

Perceived stress also mediated the relationship between self-compassion and depressive symptoms and the relationships of resilience and optimism with depressive symptoms among nursing and medical students, thus partially supporting H2. Compared with direct relationship between self-compassion and depressive symptoms, self-compassion and perceived stress have a much stronger direct relationship in this study. This result highlights the role of self-compassion in defending perceived stress [21, 31, 39]. The result in this study indicates that self-compassionate people perceive difficulties as a common humanity and tend to treat themselves kindly [30]. Thus, they are less likely to regard negative effects as heavy stress. Resilience mainly involves coping ability [46] whereas optimism involves a positive attitude [24]. Hence, they simultaneously prevent stressors and could decrease depressive symptoms by reducing perceived stress in both groups.

The model supports H1 but only partially supported H2. Resilience and optimism were directly and indirectly associated with depressive symptoms but only indirectly among medical students. This result can be explained that in the entire path model, the relationships of self-compassion with resilience, optimism, and perceived stress are stronger among medical students than that among nursing students. This condition may conceal the direct effect of resilience and optimism on depressive symptoms. This different finding may also be due to that resilient nursing students with positive attitude may be particularly equipped to manage the psychological and academic demands of nursing school [1].

Another consideration was the influence of the COVID-19 pandemic on mental health [47]. For example, the potentially high risk of exposure to infection and fear of infection were associated with high depression rates among nursing students [48]. Thus, the COVID-19’s pandemic may potentially influence the depression model, and the role of self-compassion, resilience and optimism in depressive symptoms may be enhanced in the situation. The model fit indices are acceptable. Further longitudinal studies are needed to confirm the results and explore other potential factors to improve the model fit.

### Implications from the study

Self-compassion is associated with increased resilience and optimism and decreased perceived stress, through which nursing and medical students are protected from depressive disorders. Enhancing mindfulness and kindness toward oneself and others can cultivate and improve self-compassion [49]. Thus, arranging appropriate community activities in certain courses for students to offer help to people in need, as volunteers, may help develop their loving kindness for others and for themselves.

In addition, to reduce depressive symptoms and perceived stress among nursing and medical students, self-compassion, resilience, and optimism should be enhanced simultaneously.

### Limitations

The study has several limitations. First, the cross-sectional design was the premise of causative relationships but was not sufficient for establishing a causal effect. Thus, future intervention studies are required. Second, as one of the most feasible data collection methods, self-reported measures may have response bias [50]. Nevertheless, the validated scales improve the quality of the study. Another limitation for the possibility of generalization was the cultural influence comparing with Western countries. Emotion was also influenced by culture and social situations, for culture constrains the different ways how emotions are felt and expressed in different cultural contexts [51]. Lastly, the RMSEA of both samples was more than 0.05, indicating an acceptable model fit. Thus, future studies must include other related variables to improve this model fit.

## Conclusions

Despite the limitations, the results about the role of self-compassion, resilience, and optimism shed new light on the mental health of nursing and medical students. The study findings suggested that educators and healthcare staff could incorporate training programs into the curriculum to improve the self-compassion, resilience, and optimism of students in campus and clinical environments. Teaching students with knowledge and skills and supporting their healthy growth should include self-compassion-enhancing strategies, resilience-building education, and optimism modeling in the curriculum. Optimism has largely been viewed as a personal trait [52]. However, it can change over time in changed situations [53]. The study extended that optimism can be enhanced by self-compassion in nursing and medical students.

## Data Availability

All data generated and analyzed during this study are available from the corresponding author on reasonable request.

## Abbreviations

β: Standardized estimates
Df: Degrees of freedom
CFI: Bentler’s Comparative Fit Index
CI: Confidence interval
NA: Not available
NFI: Bentler-Bonett Normed Fit Index
RMSEA: Root mean square error of approximation
SRMR: Standardized root mean square residual
TLI: Tucker–Lewis Index
